# Physical activity participation and the risk of chronic diseases among South Asian adults: a systematic review and meta-analysis

**DOI:** 10.1038/s41598-019-46154-3

**Published:** 2019-07-05

**Authors:** Susan Paudel, Alice J. Owen, Ebenezer Owusu-Addo, Ben J. Smith

**Affiliations:** 1 School of Public Health and Preventive Medicine, Monash University, Melbourne, Australia; 2 Bureau of Integrated Rural Development, Kwame Nkrumah University of Science & Technology, Kumasi, Ghana; 3 Sydney School of Public Health, The University of Sydney, Sydney, Australia

**Keywords:** Risk factors, Epidemiology

## Abstract

South Asia specific reviews on the role of physical activity (PA) domains on chronic disease prevention are lacking. This study aimed to systematically review published literature to identify the association between PA domains and chronic diseases and to provide summary estimates of the strength of association. Nine electronic databases were searched using the predefined inclusion criteria which included population (South Asian adults 40 years or older), exposure (PA or sedentary behaviour) and outcome (type 2 diabetes mellitus, breast cancer, colorectal cancer, coronary heart disease, stroke, vascular disease and musculoskeletal diseases and their markers). A random-effects meta-analysis was carried out for cardiometabolic outcomes whereas narrative synthesis was completed for other outcome variables. Inactive or less active South Asian adults were at 31% higher risk of being hypertensive. Likewise, the risk of cardiometabolic outcomes was 1.34 times higher among inactive adults. Household PA was found to have a protective effect  on breast cancer risk. Total and leisure time PA had a protective effect on osteoporosis among males and females respectively. Contemporary studies with a longitudinal design, representative samples, valid and reliable assessment of different domains are needed to establish the role of PA in chronic disease prevention in the region.

## Introduction

Chronic diseases are emerging as a public health challenge in South Asia^1,2^. Genetic predisposition, increasing life expectancy, urbanisation, mechanisation, inadequate health services and rapid economic development fuelling sedentariness and changing dietary patterns are contributing to rising chronic disease burden in the region^3–5^. Further, compared to the rest of the world, South Asians develop these diseases at a lower body mass index (BMI)^6^ and earlier in adulthood, leading to higher cost and loss of productive years^7^. Lack of awareness, poor access to health services and multiple chronic conditions further exacerbate the problem^8^.

Physical inactivity is a well-established risk factor for chronic diseases^9^. It is related to an increased incidence of coronary heart disease (CHD), type 2 diabetes mellitus (T2DM), breast cancer, colon cancer and reduced life-expectancy^10^. Compared to those who are inactive, those less active (600–3999 Metabolic equivalent (MET)-minutes) have a 14%, 16% and 3% reduced risk of T2DM, CHD and breast cancer, respectively^11^. The risk of all these diseases further decrease with higher levels of PA^11^. Overall, every 10% decrease in population-level inactivity is expected to avoid half a million global deaths annually^12^. The World Health Organization (WHO) recommends at least 150 minutes of moderate- physical activity (MPA) or equivalent per week. Increased duration to 300 minutes/week is recommended for additional health benefits^9^.

Total physical activity (PA) constitutes activities carried out across various domains of daily life, including leisure time PA (LTPA), occupational PA (OPA), household PA (HPA) and transport-related PA (TPA). Understanding all these domains is crucial as the nature of activities vary between countries^13^, and strategies to change PA will vary between the domains. LTPA is the predominant form in the high-income countries while the other three domains are more prevalent in the low and middle-income countries^14^. Consistent with other developing countries in the Asia-pacific region^13^, work and transport related activities are the most common forms of PA in South Asia^15–18^. Available data show a low prevalence of LTPA in the South Asian region: 5% among 45–59 year olds in Sri Lanka^17^, 14% among 40–69 year olds in Bhutan^16^ and 20% among 45–54 year olds in Bangladesh^18^. Variations exist both between and within the countries, with a higher prevalence of inactivity among females and urban dwellers^19,20^.

Most of the evidence regarding the effect of PA on chronic disease prevention comes from studies of leisure time activities in developed countries^21–24^. However, this domain of PA constitutes a small portion of total daily activity among South Asian adults^15–18^ and hence cannot provide an overall picture of their daily PA. Though a previous review of studies in low-and-middle-income countries by Milton *et al*.^25^ has concluded similar benefits of PA to that in high-income countries, it has also emphasised the importance of context specific research. Further, it has reinforced the importance of localised evidence to generate political support and assist in physical activity related policy development and programs^25^.

Though the demographic, epidemiological and economic transition, along with genetic susceptibility to some diseases such as T2DM, makes South Asians a priority group for chronic disease research, reviews examining the relationship between the range of PA domains and chronic diseases in the region are limited. Understanding whether the dominant forms of PA are associated with higher or lower levels of disease risk in the region is relevant to public health policy and programs. This knowledge can also provide some directions concerning the population groups and types of PA that should be the focus of determinants research. Hence, this study aimed to (1) systematically review published, peer-reviewed literature to identify the association between PA domains (total, transport, household, occupational and leisure) and selected chronic diseases and their markers, and (2) provide summary estimates of the strength of associations among South Asian adults 40 years or older.

## Methods

This systematic review has been registered with the International Prospective Register of Systematic Reviews (PROSPERO; Registration no. CRD42018096505; available from https://www.crd.york.ac.uk/prospero/display_record.php?RecordID=96505) and is guided by the Preferred Reporting Items for Systematic Reviews and Meta-Analyses (PRISMA) statement^26,27^. The review focuses on PA or sedentary behaviour in routine circumstances among South Asian adults 40 years or older. The published protocol paper further provides details of the review methodology^28^.

### Outcome measure

Chronic diseases (T2DM, breast cancer, colorectal cancer, cardiovascular disease (CHD, stroke, vascular disease) and musculoskeletal diseases (osteoarthritis, osteoporosis, back and neck pain) are the primary outcome variables while risk markers (body weight, BMI, blood sugar, blood pressure, lipids, cholesterol, bone mass density (BMD), hypertension (HTN) and the metabolic syndrome (MetS)) are the secondary outcome variables of interest. Study outcomes are classified as cardiometabolic conditions (HTN, T2DM, MetS or CHD), breast cancer and musculoskeletal conditions for reporting the review results.

### Search strategy and inclusion criteria

Nine electronic databases: MEDLINE, EMBASE, PSYCINFO, CENTRAL, CINAHL PLUS, SPORTDiscus, AgeLine, Scopus and the Web of Science were systematically searched for English language, peer-reviewed papers published between January 2000 and March 2018. The MEDLINE search strategy was developed through a review of published literature and in consultation with a medical librarian experienced in systematic reviews. It was then adapted to other databases with an additional limit of excluding MEDLINE records whenever the databases provided that option. The MEDLINE search strategy is presented in Supplementary File: Table 1. A manual search of references and forward citations of relevant systematic reviews and relevant articles was also carried out to ensure all potential studies were captured.

The search was limited to quantitative studies examining the association between chronic disease or their risk markers and PA in routine circumstances among South Asian adults 40 years or older. In this review, routine PA refers to activities of varied intensities carried out as part of a regular daily routine and can relate to regular work, household, transport or leisure-time activities. Structured activities carried out in a controlled, and supervised environment for research purposes were excluded. No limits were applied for study design, but non-peer reviewed literature was excluded.

### Study selection

All the identified articles were initially imported into Endnote X8 software^29^ and duplicate records were removed. These articles were then uploaded to Covidence systematic review software (available at www.covidence.org) where SP screened the titles. Two reviewers (EOA and SP) then independently screened each abstract and full text article against the predefined inclusion/exclusion criteria. Only those records which were included by both the reviewers passed on to the final review stage. Discrepancies were resolved by discussion and consensus among the authors. Reference lists of these eligible studies were manually checked to ensure no potentially relevant articles were missed.

### Data extraction and quality assessment

A Microsoft Excel data extraction template was developed, pretested and approved by the review team before data extraction. SP then extracted the data using this template while EOA verified the accuracy and completeness of the extracted data. Information on authors, publication date, country of origin, study population, sample size, outcome measures, exposure variables, types of PA, measures of association and the study findings were extracted from all eligible studies. Several study authors were contacted to clarify study details. The review utilised information reported in the articles when the study authors did not respond.

The National Institute of Health (NIH) quality assessment checklist was used to assess the quality of the included studies with separate checklists for case-control and cross-sectional studies^30^. Included studies were assessed against several quality criteria such as research question, study population, participation rate, inclusion criteria, sample size, exposure prior to outcome, sufficiency of time frame, different levels of exposure, exposure measures, multiple exposure assessment, outcome measures, blinding, follow-up rate and statistical analyses^30^. SP and EOA independently assessed the quality of the papers. Final quality scores were assigned to the studies in consultation with BJS and AJO. A score higher than 75% was considered good quality, 50–75% was deemed as fair quality and less than 50% as poor quality as used in previous studies^31^. The review team was not blinded to the authors or journals during the review process.

The quality of evidence for each outcome measure was assessed using the Grading of Recommendations, Assessment, Development and Evaluation (GRADE) framework^32^. GRADE provides four categories: high, moderate, low and very low for evidence grading. Higher quality indicates greater confidence that future research is unlikely to change the effect estimate while lower quality indicates higher likelihood that future research will change the effect estimate and the level of confidence in the estimates. The framework allows for upgrading or downgrading the quality of evidence depending upon the risk of bias, imprecision, indirectness or inconsistency^32^. SP evaluated the overall quality of evidence for each study design which was then verified by the review team.

### Statistical analysis

A General variance-based random effects modelling was used to calculate summary Odds Ratios (OR) and 95% Confidence Intervals (CI) for PA and cardio-metabolic outcomes. When a single study reported more than one outcome^33,34^, only the primary outcome variable was used to avoid the issues of non-independent data^35^. In this analysis, the highest PA category was compared with the lowest PA category, using the highest category as the reference group. When PA was categorised into more than two categories, the highest and lowest categories were compared. Adjusted estimates were used wherever available. Out of 9 studies, reported ORs were used for pooling in 6 instances^33,36–40^, three of which required the reference category to be reversed^38–40^, with ORs calculated from reported raw data in the remaining three studies^34,41,42^. We did not convert the correlation coefficients from cross-sectional studies to ORs due to the substantial difference in the nature of studies reporting these two different measures of association^35^.

Sub-group analysis was performed across cardio-metabolic outcomes (HTN, T2DM, CHD and MetS), study design (cross-sectional vs case-control studies) and country (India vs non-India based papers). Sensitivity analysis was conducted by removing one study at a time to determine the impact this had upon pooled results. I^2^ and Chi2 values were calculated to test the magnitude of heterogeneity. Publication bias was assessed using a funnel plot of standard error versus effect size and Egger’s test. All statistical analyses were performed using Review Manager version 5 software (RevMan 5)^43^. Whenever a meta-analysis was not feasible because of a limited number of studies or heterogeneity across the studies, a narrative summary was produced.

## Results

### Study selection

**Figure 1:**
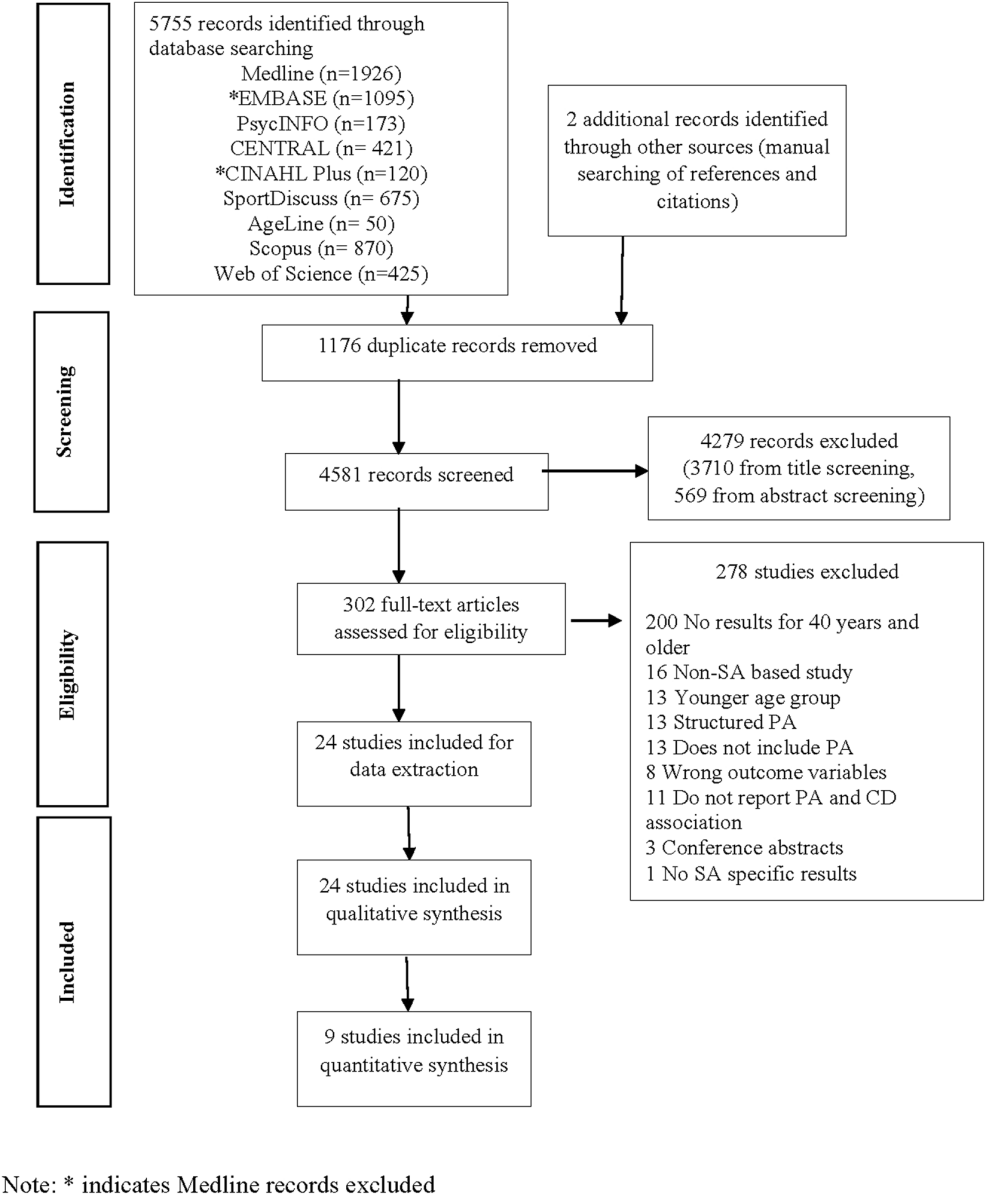
Flowchart of study selection.

A systematic search of nine electronic databases identified 5755 records while two additional articles were located from manual searching of references and citations. The 4581 non-duplicate records were then screened for title and abstracts which further excluded 4279 records: 3710 from title screening and 569 from abstract screening. Full texts of the remaining 302 articles were retrieved and assessed in detail using predefined criteria, and an additional 278 records were excluded in this stage. The majority (72%) of the articles were excluded because the studies did not report associations between PA and chronic diseases/risk factors for people 40 years and older. Figure 1 presents the study selection process using the PRISMA flow chart.

There were 24 studies included in the narrative synthesis, 9 of which were also included in the quantitative synthesis of the relationship between PA and cardio-metabolic outcomes. Seven of the 16 studies examining cardio-metabolic outcomes could not be included in the pooled analysis for one or more of the following reasons: they did not report any effect estimate or provide convertible raw data^44,45^; the effect estimate was not given in a form that could be transformed and included (such as hazard ratio^46^ or correlation coefficient^47^); or they reported PA on a scale that did not allow us to combine the results with other studies^48,49^. A case-control study by Kumar^50^ provided raw data for the relationship between PA and a secondary outcome variable, but the calculated OR was markedly higher than those reported in other studies and hence was not used. Meta-analysis of other outcome variables (i.e. musculoskeletal conditions, breast cancer) was not feasible because of a limited number of studies and heterogeneity regarding PA types and their categorisation.

### Study characteristics

Of the 24 studies included in this review, more than half (54%) were conducted in India^36,38,40,42,44,45,47–49,51–54^, two each in Pakistan^34,46^ and Nepal^33,41^ and one each in Sri Lanka^55^, Bangladesh^56^ and Afghanistan^37^. One study^57^ reported results for five South Asian countries while two studies^39,50^ were based in two South Asian countries. More than half of the studies (14 out of 24) were published between 2001 and 2010. Eighteen of the 24 studies were cross-sectional in design^33,37–42,44–49,53–57^ while the remaining six were matched case-control studies^34,36,50–52,58^. Age was one of the matching variables in all these six studies. Six studies examined women only^49,51,52,54,56,58^, four examined men only^36,48,53,55^ and two reported results separately for men and women^46,57^. Supplementary File: Table 2 summarises the descriptive characteristics of the included studies.

Eight of the studies reported response rates, which was more than 80% in all cases^38,39,45,46,49,51,52,56^. A sample size justification or a power calculation was provided only in one-third of the included studies^33,37,39,41,44,49,53,54^. Altogether, this review includes results from 26,092 participants with the sample size in the cross-sectional studies ranging from 90^48^ to 7238^38^ and between 330^50^ to 1659^52^ in the case-control studies. One study^45^ did not report a sample size for the target age-group and hence was not included in total sample size calculation for this review.

The majority of the studies (75%) analysed the association between PA and chronic diseases or their risk factors using regression analysis, although three reported p-values from a chi-square test^41,42,55^, one reported a p-value from a t-test^50^, and two reported correlation coefficients^47,48^. The presence of chronic diseases or their risk factors were established through medical records, biochemical tests (such as an oral glucose tolerance test (OGTT), blood tests), anthropometric measurements and bone scans. Two studies relied on patients self-report of their conditions^42,57^.

**Figure 2:**
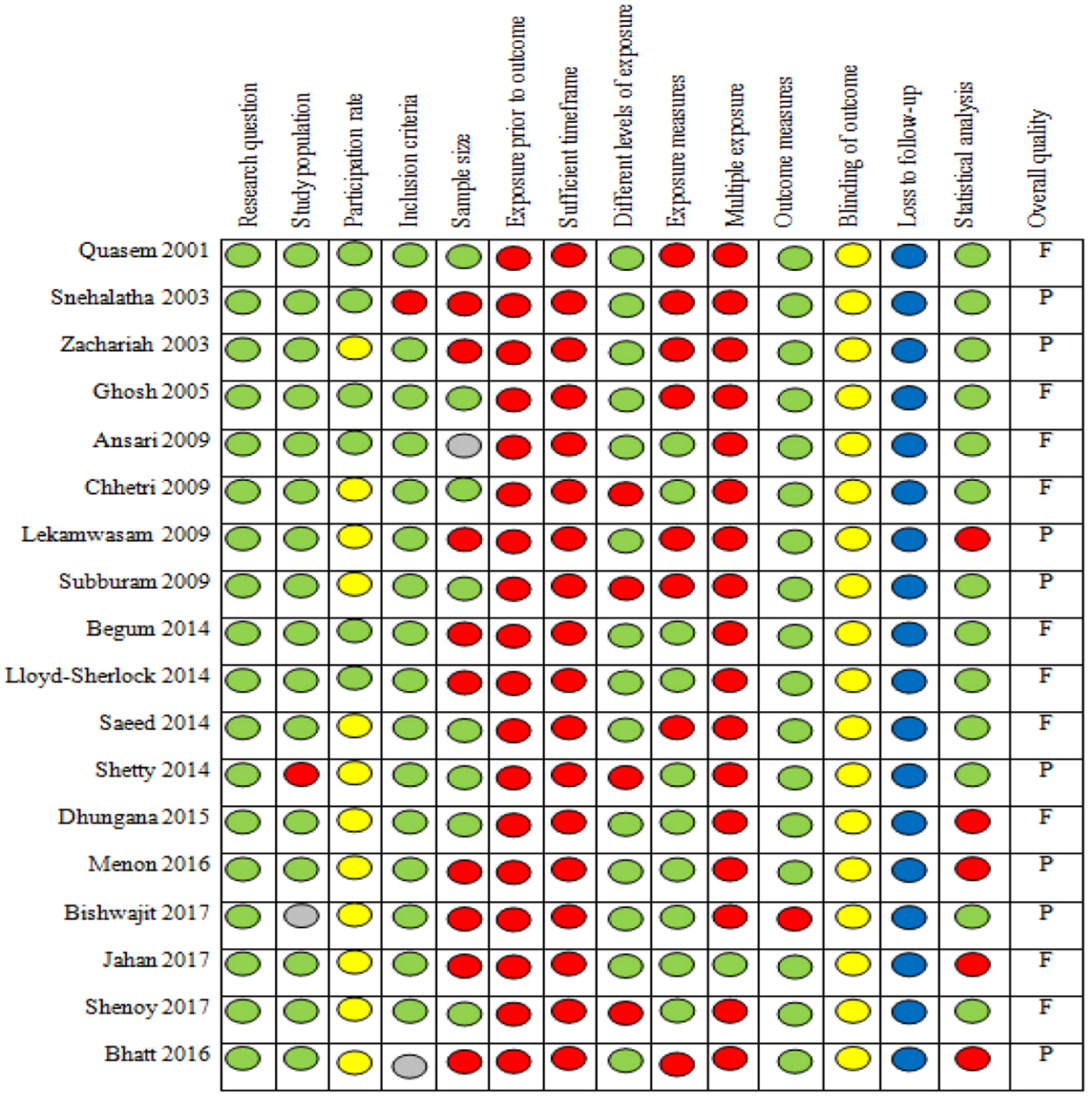
Quality assessment of cross-sectional studies using the NIH checklist.

Application of the quality assessment checklists showed that the quality scores ranged between 32% and 62% for cross-sectional studies and between 42% and 58% for case-control studies. None of the 24 studies was ranked as high quality, 11 were of fair quality, and the remaining 13 were of poor quality. Figure 2 shows the quality ranking of cross-sectional studies across different criteria with green circles for “yes”, red for “no” and yellow, grey and blue circles for “not reported”, “cannot determine” and “not applicable” respectively. The quality of evidence from all studies for all outcome measures was rated “very low” based on the GRADE framework (Table 1). The quality was downgraded from “low” to “very low” because of serious risk of bias (such as questionable validity and reliability of the measurement instrument, no sample size justification or recruitment of cases and controls from different populations) and/or serious imprecision which implies that future studies are highly likely to change the estimate and/or the level of confidence in the estimates.

**Table 1:** Overall summary of findings by outcome measures.

Outcome measures	Number of studies	Quality of evidence^a^	Summary of findings (number of studies reporting direction of association across the PA domains)^b^
**Cardiometabolic outcomes**			
HTN	5	Very low	Total PA: null (1), mixed (1) OPA: null (1), mixed (1) Walking: null (1)
T2DM	3	Very low	Total PA: null (1), mixed (1) LTPA: mixed (2) Walking, cycling: null (1)
CHD/CVD risk	3	Very low	Total PA: null (2), negative (1)
Obesity measures	5	Very low	Total PA: negative (2) Walking: null (2), negative (1)
Breast cancer	2	Very low	HPA (5–6 hr/day): negative (2) Walking: mixed (1) Watching television: null (1)
**Musculoskeletal conditions**			
Osteoporosis	5	Very low	Total PA: null (1), negative (2) LTPA: null (1), negative (1) Walking: null (1)
Back pain	1	Very low	Walking, moderate and vigorous total PA: mixed (1)

^a^The quality of evidence is assessed using the GRADE criteria and has been downgraded from “low” to “very low” for all the studies because of the serious risk of bias and/or serious imprecision indicating that future studies are highly likely to change the estimate and/or the level of confidence in the estimates.

^b^The number of studies reporting null/mixed associations will not add up to the total number of studies for an outcome measure as some studies have reported more than one domain of PA.

### PA domains and assessment

There was considerable variation in the domains of PA studied. Most of the included studies reported associations between total PA and the outcome variables^34,36,38,39,41,42,45–47,53,55–57^. Nine studies reported associations for walking^37,46–50,52,57,58^, four reported LTPA^33,46,54,58^, three reported HPA^46,51,52^ and two reported OPA^40,44^. All studies, except one^47^, used questionnaires to measure PA. The study by Jahan and Shenoy^47^ used both the International Physical Activity Questionnaire (IPAQ) and pedometer counts to determine participants’ PA scores^47^. None of the case-control studies used a standardised instrument to measure PA or reported any form of reliability and validity estimates for the measurement tool used. On the other hand, 10 of the 18 cross-sectional studies either used previously validated questionnaires such as IPAQ^47,54,56^, the Global Physical Activity Questionnaire (GPAQ)^38,41,42^, or another instrument for which validity and/or reliability was reported^33,46,53,57^. The majority of the studies collected information regarding activities carried out in the last seven days or an average day/week^36,39–41,44,47,49,51,52,54–57^ while 10 studies (42%) did not provide any information on the recall period^33,34,37,38,42,45,48,50,53,58^. A study by Ansari^46^ collected information regarding engagement in exercises and sports during the last two years while another study concerning breast cancer asked participants to report the activities carried out one year before the disease was diagnosed^52^.

### Association between PA and chronic diseases

#### Cardio-metabolic outcomes

The associations between PA and cardio-metabolic outcomes were examined in 16 of the 24 studies. Of these 16 studies, 5 reported HTN^37–40,44^, 3 each reported T2DM^33,45,46^ and CHD or CVD risk^36,41,42^ and 1 reported metabolic syndrome^34^. The remaining studies examined body composition measures (such as body weight, fat mass index (FMI), fat-free mass index (FFMI), waist circumference (WC), waist-hip ratio (WHR)) or total cholesterol as the outcome variables. Among these studies, two were specific to males^36,48^, one was specific to females^49^ while one reported associations separately for males and females^46^. Several studies were limited to one type of PA while some examined associations across multiple PA types. All studies, except two^36,50^, were cross-sectional in design.

Among the five studies examining the association between PA and HTN, 1 reported negative association^39^, 2 reported null associations^37,44^ and 2 reported mixed associations^38,40^. The mixed associations were reported in 2 studies which examined occupational PA and HTN^40,44^. Zachariah *et al*.^40^ found a decreased likelihood of HTN among individuals whose occupation involved MPA (AOR: 0.35 (0.13–0.94)) compared to those with sedentary occupations while there was no significant effect of mild PA. On the other hand, Subburam *et al*. reported no significant difference in the risk of HTN among individuals whose occupations involved a varying degree of PA^44^.

Of the three studies reporting T2DM, one each found a negative^33^, null^45^ and mixed associations^46^, respectively. Both the studies reporting associations between LTPA and T2DM found an increased risk of diabetes among individuals with low activity levels^33,46^. Stair climbing and cycling were also inversely associated with the risk of diabetes, but there was no effect of HPA^46^.

Two of the three studies reporting associations between total PA and CHD or CVD risk found null association^36,41^ while the other reported negative association^42^. Mixed associations were reported for blood pressure^37,47,50^ and fat mass^48,49^ and a negative association was reported with cholesterol^50^.

Overall, among 16 studies reporting cardiometabolic outcomes, null associations were reported in 5 of the 16 studies^36,37,41,44,45^, negative associations in another 5 studies^34,39,42,49,50^ and mixed associations were reported in the remaining 6 of the16 studies^33,38,40,46–48^. One-quarter of the included studies (6 of the 24) reported associations between walking and cardio-metabolic conditions. No association existed between walking and T2DM^46^, waist circumference^47,48^ and BMI^48^. Supplementary File: Table 3 provides study specific associations.

**Figure 3:**
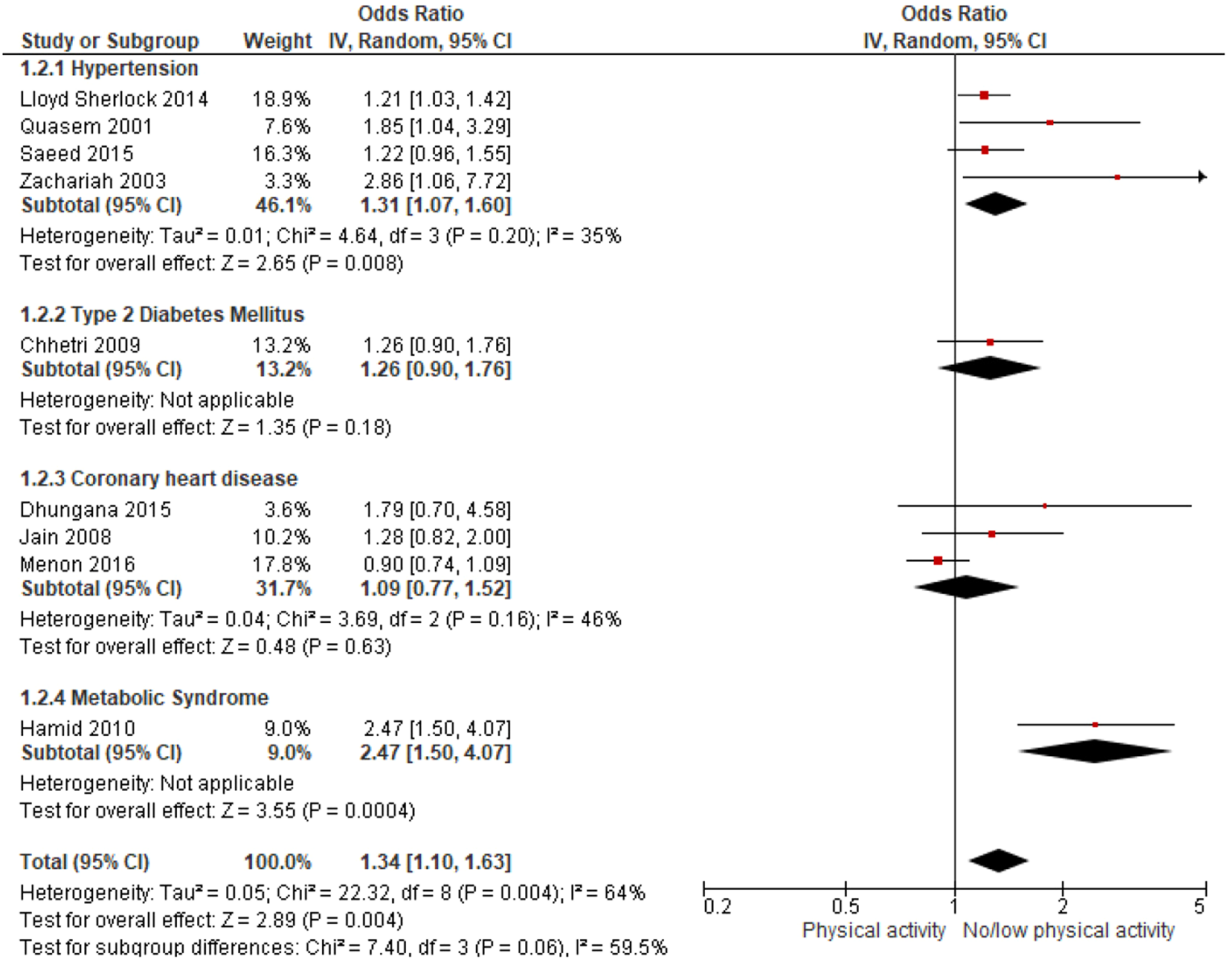
ORs of cardiometabolic outcomes for physically active versus inactive individuals. Horizontal bars represent confidence intervals and small squares represent relative contribution of each study in pooling. An OR > 1.00 indicates higher odds of cardiometabolic outcomes among inactive individuals.

**Figure 4:**
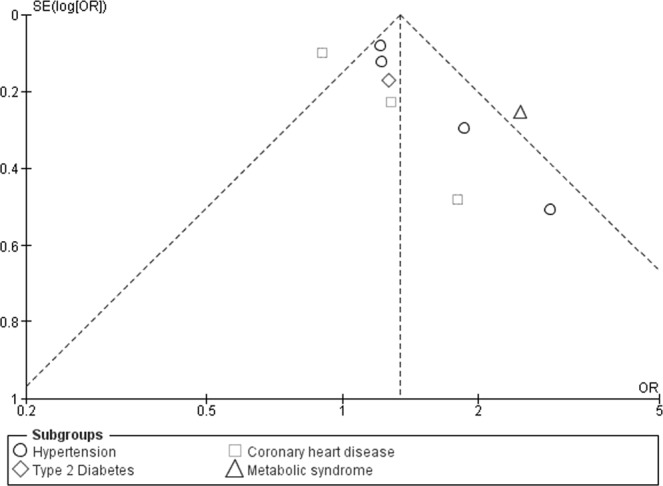
Funnel plot for PA and cardiometabolic outcomes.

The results from the meta-analysis of 9 studies showed an inverse association between PA and cardiometabolic outcomes. The pooled OR for HTN was 1.31 (1.07–1.60) indicating that the inactive or those with low levels of PA were 31% more likely to be hypertensive (Fig. 3). No statistically significant association was found between PA and CHD or CVD risk (pooled OR: 1.09 (0.77–1.52), I^2^ = 46%). Overall, South Asian adults with no or low PA were 1.34 times more likely to suffer from cardio-metabolic conditions than active adults (pooled OR: 1.34 (1.10–1.63), I^2^ = 64%) ((Fig. 3). The pooled result for cross-sectional studies resulted in an OR of 1.23 (95% CI: 1.02 to 1.48) while pooled OR of case-control studies was 1.76 (95% CI: 0.92–3.35) (figure not shown). Pooled OR for studies conducted in South Asian countries other than India indicated a 49% higher risk (pooled OR: 1.20 (1.06–1.59), I^2^ = 65%) of cardiometabolic outcomes among inactive individuals, however, the pooled OR was not significant for studies conducted in India (figure not shown). Sensitivity analysis was performed by removing one study or one disease group at a time, but this had no substantial effect on the pooled effect size (results not shown). The funnel plot showed some evidence of publication bias (Fig. 4). This is further confirmed by Egger’s test (p-value: 0.032).

### Breast cancer

The relationship between breast cancer and PA was examined in 2 of the 24 studies^51,52^. Both studies were age and residence status (urban/rural) matched case-control studies among post-menopausal Indian women. Cases were histologically confirmed incident primary breast cancer cases while controls were cancer-free women who accompanied another type of cancer patient to the same hospital. The study by Dey further classified the cases by estrogen receptor (ER) status and reported associations separately for ER + and ER- cases^51^.

Both studies reported decreased breast cancer risk among postmenopausal women engaged in HPA^51,52^ (Supplementary File: Table 4). Dey *et al*. found a protective effect of increased duration of HPA for both ER + (p-value for trend = 0.003) and ER- cases (p-value for trend = 0.009)^51^. Postmenopausal women engaging in HPA for 5–6 hours/day were 40% less likely to have ER + breast cancer (AOR: 0.60 (0.36–0.98) than women undertaking <3 hours/day of HPA. The study found no protective effect of engaging in HPA for 3–4 hours/day and >6 hours per day for both ER + and ER- cases^51^. Mathew *et al*. reported 51% and 49% decreased risk of breast cancer among women engaging in 5–6 hours and more than 6 hours/day of HPA, respectively^52^. Watching television during the weekdays or weekends was not associated with breast cancer risk among postmenopausal women^52^. Study-specific results are summarised in Supplementary File: Table 4: Characteristics and results of studies: Breast Cancer.

### Musculoskeletal conditions

Six of the included 24 studies (25%) examined the association between PA and musculoskeletal conditions (Supplementary File: Table 5). Four cross-sectional studies^53–56^ and one case-control study^58^ reported associations between PA and osteoporosis while another cross-sectional study^57^ reported associations for self-reported back pain. Three studies were limited to females^54,56,58^ and the remaining two were specific to males 50 years or older^53,55^.

### Osteoporosis

Both studies which examined associations between total PA and osteoporosis among males found a protective effect^53,55^. Shetty *et al*. reported 40% less risk (adjusted OR: 0.4 (0.12–0.9), p < 0.0001) of osteoporosis among physically active males compared to their inactive counterparts^53^.

Among females, one study reported no association between osteoporosis and total PA^56^ while two studies found a protective effect of LTPA^54,58^. There was 32% less risk of osteoporosis with every additional 10 metabolic equivalents (METs) of LTPA (adjusted OR: 0.68 (0.66–0.71))^54^. Only one study examined the association between regular walking and BMD and found a null association^58^.

### Back pain

The association between back pain and PA was examined in one study that was conducted across five South Asian countries^57^. Bishwajit *et al*. analysed the data from the World Health Survey 2002 among 8502 men and women aged 50 years and above from Bangladesh, India, Nepal, Pakistan and Sri Lanka^57^. Separate associations were reported for walking, MPA, and vigorous PA (VPA).

Walking was not found to have a significant association with back-pain among women except in India. Indian women who walked a few days/week or who never walked had 26% (AOR: 1.26 (1.00–1.58)) and 33% higher odds (AOR: 1.33 (1.00–1.75)) respectively of reporting back pain compared to women who walked daily. In the case of men, similar associations were found in Nepal and Pakistan but only for those who never walked^57^.

Indian men and women who did not engage in daily MPA were more likely to report back pain. For those who did not undertake any MPA, the odds of reporting back pain were 29% (AOR: 1.29 (1.04–1.59)) and 56% (AOR: 1.56 (1.00–2.44) higher for women and men respectively, compared to those undertaking daily MPA. Likewise, Indian men and women engaging in MPA for a few days a week were 38% and 36% more likely to report back-pain than those undertaking daily MPA. No significant associations were found for Bangladeshi and Nepali men and women and Sri Lankan men. In case of Pakistani and Sri Lankan women, a higher risk was found among those undertaking no MPA. The study did not find any significant association between VPA and self-reported back pain among men across all five countries. Significant associations were found among women in Pakistan and Sri Lanka^57^. Supplementary File: Table 5 presents country-specific findings.

## Discussion

This study systematically reviewed 24 peer-reviewed studies to determine the association between PA and chronic diseases among South Asian adults aged 40 years and older. Total PA was the most reported exposure variable, with few studies reporting other PA forms such as walking, HPA or LTPA. Cardiometabolic outcomes were the most studied outcome variables, followed by musculoskeletal conditions. No clear dose-response relationship was evident because of differences in PA classifications and domains, and mixed associations across levels of PA categories.

### PA and cardiometabolic outcomes

The results from the meta-analysis indicate an increased risk of cardio-metabolic outcomes (HTN, T2DM, MetS or CHD) among inactive South Asian adults. The risk was 34% (range, 10–63%) higher among inactive people compared to those with moderate or higher levels of total PA. Some of the studies could not be pooled because they did not report ORs, provide raw data or other convertible effect estimates, or they used PA categorisations that were not comparable to other studies. This reflects the heterogeneity across the studies and highlights the need to use comparable PA measures and classifications in future studies.

Inactive or less active South Asian adults were at 31% (range, 7–60%) higher risk of being hypertensive. The results are consistent with the existing literature, which has found that PA is a preventive, as well as a treatment strategy for managing HTN^59^. A meta-analysis of non-South Asia based cohort studies has reported a 41% increased risk (range, 15–72%) of HTN among individuals with low total PA compared with those with high total PA^60^. Another meta-analysis reported no association between OPA and HTN^61^, however, our review found mixed results across the two studies reporting OPA. Hypertension is an escalating public health problem among South Asian adults with the prevalence ranging from 20% in Bhutan (40–69 years)^16^ to 24% in the Maldives (45–64 years)^62^ and 47% in Nepal (45–69 years)^15^. The findings of this meta-analysis indicate the potential value of public health interventions promoting total PA to reduce the burden of HTN in South Asia.

Engagement in LTPA was found to have a protective effect on T2DM risk, as reported in 2 studies, however, this review concluded that there is limited evidence on the role of PA on T2DM risk among South Asian adults because of the small number of studies reporting the association. A meta-analysis of cohort studies including at least 2 of the 4 PA domains has reported a decreased risk of T2DM incidence by 26% among individuals with at least 150 min/week of MPA^63^. This protective effect of PA on T2DM has been reported in other studies^25,64^. Further studies are required to establish the role of PA in tackling the T2DM burden in the South Asian region.

There was no statistically significant association between CHD or CVD risk and total PA in this review. This finding contrasts with other reviews that have reported a protective effect^11,25,65^. The Global Burden of Disease Study 2013 found a reduced CHD risk of 25% and 23% among highly and moderately active individuals respectively, compared to those insufficiently active^11^. The plausible explanation for the differences in findings could be the nature of the studies included in our meta-analysis. Of the three studies used for pooling, one was a case-control study among CHD cases^36^ while the other two papers used different CVD risk scores^41,42^. Future studies using standard outcome measure (CHD incidence) and PA assessment criteria are recommended to determine the role of PA in CHD prevention in the region.

While non-South Asia based studies show a negative association between PA and obesity^66–68^, the only study included in this review found no significant correlation between PA and BMI, WC, WHR and MetS^47^. Walking was also not found to be associated with decreased waist circumference or BMI. These findings need to be interpreted with caution as they are derived from a few studies only. Additionally, lack of objective assessment of PA in most of the studies might have resulted in an under or over reporting of levels of activity and underestimation of its association with weight and metabolic variables^69^.

### PA and breast cancer

Both the studies reporting an association for breast cancer found a protective effect of HPA among Indian women, with 5–6 hours/day of HPA being the optimum amount. This finding is consistent with systematic reviews on breast cancer and HPA. A meta-analysis that pooled 21 HPA comparisons from 15 studies found that the risk of breast cancer was reduced by 22% among those with the highest HPA level compared to the lowest^70^. Another meta-analysis has revealed a risk reduction of 11% (95% CI: 5% to 17%) for HPA^71^. The findings of the current review are particularly crucial in the South Asian context where HPA is the dominant PA form among women^15,16,19,62^. However, the findings need to be validated with larger longitudinal studies.

### PA and musculoskeletal conditions

Adults are at an increased risk of osteoporosis because of physical inactivity, morbidities, hormonal changes and decreased intake of calcium and vitamin D^72^. It is an increasing problem in South Asia, where there is often late diagnosis, and is reported to be exacerbated by vitamin D deficiency^73–75^. In India, it has been reported that hip fractures due to low BMD occur almost a decade earlier compared to the western nations^74^. Our review found that total PA had a protective effect on osteoporosis among males only.

On the other hand, LTPA had a protective association among females, but no association was found for walking. A review of intervention studies has found that PA prevents bone loss and has a protective effect on BMD among postmenopausal women, but was unable to conclude the type, intensity, duration and frequency of PA that is beneficial^72^. Because of a limited number of studies eligible in our review, it is difficult to reach to a definite conclusion about the role of PA in the prevention of osteoporosis in South Asia.

The only study that examined the association between self-reported back pain and PA, across five South Asian countries, found mixed associations across PA type and dosage between males and females. Previous systematic reviews have also found an inconsistent association between low back pain and PA^76,77^. A review of systematic reviews concluded limited evidence on the causal relationship between walking and low back pain^78^. Given the limited research on back pain and different PA domains, and the inconsistency across the available evidence, further research is needed to reach to a definite conclusion in the South Asian context.

### Methodological limitations of the included studies

The lack of age-specific results was the primary reason for exclusion of more than 70% of screened papers for this review. All the studies were either ranked as fair or poor quality using the NIH checklist. Common weaknesses were the lack of temporal difference in exposure and outcome measurement, insufficient timeframe for the outcome to manifest, not having repeated exposure measurement, and not using validated measurement instruments. All studies, except one, used questionnaires to assess the participant’s PA. While questionnaires are the measures of choice in population-level surveys, particularly in developing countries to capture activities across the range of PA domains^79,80^, it cannot be denied that they are likely to under or overestimate correct exposure because of recall or social desirability bias or the lack of common understanding between respondents and researchers^81^.

Meta-analysis was only possible for nine studies reporting cardio-metabolic outcomes because of the heterogeneity of outcome variables, PA domains, and availability of raw data or convertible measures of association. The recall period was not mentioned in 10 of the 24 studies while one study asked participants to recall the activities carried out during the last two years^46^. Only one-third of the studies reported a response rate and sample size calculation, which made it difficult to ascertain the representativeness of the sample. Poor reporting of research methods was common in the included articles.

The studies included in this review have shown either a null association, negative association or a mix of null and negative association across the PA domains/categories. While some of these differences might be real, the variations in the type of PA questionnaires used, types of PA domains studied and the categorisation of PA scores for reporting of the results might have affected the study results. Comparing the results across the studies was difficult because of the variations in the categorisation of PA between the studies. Using the standard categorisation of PA based on the cut-offs suggested in the GPAQ and IPAQ analysis guides^82,83^ would help to maintain uniformity and facilitate comparison between studies. Longitudinal studies with relatively larger sample size, use of validated tools for PA assessment and objective assessment of chronic diseases are recommended to ensure the production of high-quality evidence to inform policy and practice.

### Strengths and limitations of the review

To our knowledge, this is the first study to systematically review the association between PA and multiple chronic disease outcomes and risk markers and to quantitatively summarise the association between cardiometabolic outcomes and PA among South Asian adults. Routine PA was the primary focus of the review and studies which only examined structured PA were excluded, which was a strength given that routine HPA and TPA contribute to the significant portion of PA among adults in South Asian nations^15–18^.

Several limitations also need to be considered when interpreting the study findings. More than half of the included studies were from India while none were conducted in the Maldives or Bhutan. Two-thirds of the studies were cross-sectional which restricts the ascertainment of causality. Meta-analysis dichotomised PA as yes/no or low/high because of the lack of estimates for the middle category in some studies, which decreased the sample size in the pooled analysis and increased the confidence intervals for the odds ratios. Further, only statistical significance, not clinical significance, was considered while interpreting the study findings. Limiting the search only to published peer-reviewed English language studies could have missed some information.

## Conclusion

The rapidly changing demographics, haphazard urbanisation and economic development, along with genetic susceptibility to diseases such as T2DM, make South Asians a priority group for non-communicable disease risk factor research. The pooled results from the meta-analysis of observational studies included in this review suggest that physical inactivity is associated with the higher risk of cardiometabolic conditions, particularly, hypertension among South Asian adults. Public health interventions addressing PA could potentially contribute to addressing the surging cardiometabolic disease burden in the region. The limited number of studies included in this review restricted drawing conclusions on the association of PA with other outcome variables such as T2DM, osteoporosis, CHD, obesity and breast cancer. Based on the global evidence, this review also recommends incorporating PA in interventions targeting these conditions along with conducting high-quality studies, with a longitudinal design, representative samples, and objective assessment of PA and disease outcomes, to generate local evidence. Scientific evaluation of existing interventions can also provide useful information on the role of PA in chronic disease prevention. Further, most of the studies included in this review have examined total PA with very few that investigated associations for different PA domains: including occupational, household and transport related PA. Future studies should focus on all the PA domains and use standard categorisation of PA to allow for comparisons across the studies.

## Supplementary information

Supplementary file

## Data Availability

This review uses data and findings from already published studies that are publicly available.
