# Supplementary material for: Physical activity participation and the risk of chronic diseases among South Asian adults: a systematic review and meta-analysis
Source: Sci Rep. 2019 Jul 5;9:9771. doi: 10.1038/s41598-019-46154-3 (PMC6611898; doi:10.1038/s41598-019-46154-3)
Supplement: Supplementary file 1 — Supplementary file [file 41598_2019_46154_MOESM1_ESM.pdf]

Physical activity participation and the risk of chronic diseases among South Asian adults: a systematic review and meta-analysis

Authors: Susan Paudel[1*], Alice J. Owen[1], Ebenezer Owusu-Addo[1, 2], Ben J. Smith[1,3]

Authors' information:

[1]School of Public Health and Preventive Medicine, Monash University, Melbourne, Australia.

[2]Bureau of Integrated Rural Development, Kwame Nkrumah University of Science & Technology, Kumasi, Ghana.

[3]Sydney School of Public Health, The University of Sydney, Sydney, Australia.

*Correspondence: susan.paudelsubedi@monash.edu, replysusan@gmail.com

Table 1: Search strategy in Medline database

Title of the database searched: **Ovid MEDLINE(R) Epub Ahead of Print, In-Process & Other Non-Indexed Citations, Ovid MEDLINE(R) Daily, Ovid MEDLINE and Versions(R)** 1946 to March 14, 2018
Name of the software platform: Ovid
Date search was run: 18 March 2018
Limits used: English language, humans, age group: 19+, journal articles
Search Strategy:

#	Searches	Results
1	bangladesh/ or bhutan/ or india/ or nepal/ or pakistan/ or sri lanka/	123041
2	(bangladesh* or bhutan* or india* or afghan* or nepal* or pakistan* or sri lanka* or srilanka* or maldiv* or south asia* or southasia*).mp.	243160
3	1 or 2	243160
4	exercise/ or muscle stretching exercises/ or resistance training/ or running/ or jogging/ or swimming/ or walking/ or stair climbing/ or leisure activities/ or recreation/ or dancing/ or gardening/ or games, recreational/ or video games/ or sports/ or physical fitness/ or bicycling/	209480
5	(physical activit* or physical inactiv* or exercise* or nonexerciser* or non exerciser* or muscle stretching exercise* or resistance train* or run* or jog* or swim* or walk* or stair* climb* or leisure activit* or recreation or danc* or gardening or games, recreational or sport* or physical fitness* or cycle* or bicycl*).mp.	1287470
6	"Physical Education and Training"/ or Motor Activity/ or life style/ or sedentary lifestyle/	156874
7	(physical education or fitness* or motor activity).mp.	185211
8	(lifestyle* or life style* or sitting time).mp.	121344
9	(sedentary adj1 (lifestyle* or activit* or behavio?r* or time)).mp.	12706
10	TELEVISION/ or Computers, Handheld/ or SMARTPHONE/ or Cell Phone/	24081
11	(computer* or smartphone* or mobile phone* or cell phone* or television* or TV).mp.	768086
12	(leisure adj3 activit*).mp.	12667
13	((household or domestic) adj activit*).mp.	792
14	(leisure adj2 exercise*).mp.	449
15	((TV or television) adj3 (watch* or view*or time)).mp.	2729
16	((screen or screen based) adj2 (time or view*)).mp.	1857
17	4 or 5 or 6 or 7 or 8 or 9 or 10 or 11 or 12 or 13 or 14 or 15 or 16	2188959
18	diabetes mellitus, type 2/	111829
19	chronic disease/ or noncommunicable diseases/	246642
20	(chronic disease* or noncommunicable disease* or non communicable disease*).mp.	291459
21	((type II or type 2) adj diabetes).mp.	108488
22	(T2D or NIDDM or noninsulin or non insulin).mp.	21904
23	coronary disease/ or coronary artery disease/ or myocardial infarction/	313408

24	stroke/ or brain infarction/	85134
25	Breast Neoplasms/ or colorectal neoplasms/ or musculoskeletal diseases/	338017
26	((breast or colon or colorectal) adj3 (cancer* or tumo?r* or neoplasm*)).mp.	476115
27	(coronary disease* or coronary artery disease* or myocardial infarction or myocardial isch?emia or stroke* or cerebrovascular infarction or cerebrovascular accident or musculoskeletal disease* or brain infarction).mp.	656045
28	((coronary or heart) adj2 disease*).mp.	408235
29	Vascular Diseases/ or Peripheral Vascular Diseases/ or Peripheral Arterial Disease/	48804
30	(vascular disease* or peripheral vascular disease* or peripheral arter* disease*).mp.	86761
31	LOW BACK PAIN/ or BACK PAIN/ or NECK PAIN/	39444
32	OSTEOARTHRITIS/	33363
33	OSTEOPOROSIS, POSTMENOPAUSAL/ or OSTEOPOROSIS/	50905
34	(low back pain or neck pain or back pain or osteoarthritis or osteoporosis).mp.	209731
35	18 or 19 or 20 or 21 or 22 or 23 or 24 or 25 or 26 or 27 or 28 or 29 or 30 or 31 or 32 or 33 or 34	1951435
36	body weight/ or overweight/ or obesity/ or adiposity/ or body mass index/ or waist-hip ratio/	388880
37	(body weight or overweight or obes* or adipos* or body mass index or BMI or waist-hip ratio or waist circumference* or waist-height ratio).mp.	778557
38	((abdominal or body) adj2 fat).mp.	44115
39	Blood Pressure/ or blood glucose/ or lipids/ or triglycerides/ or metabolic syndrome/ or waist circumference/ or waist-height ratio/	559868
40	HYPERTENSION/ or hyperglycemia/ or glucose intolerance/ or hyperinsulinism/ or insulin resistance/ or CHOLESTEROL, LDL/ or CHOLESTEROL/ or CHOLESTEROL, HDL/	428054
41	(blood adj (glucose or sugar* or pressure)).mp.	575970
42	(lipid* or triglycer* or hypertensi* or hyperglycemi* or glucose intoleran* or hyperinsulin* or insulin resistan* or cholesterol* or fatty acid* or fatty tissue* or glyc?emic index or hypercholesterol* or metabolic syndrome).mp.	1384690
43	(pressure adj (diastolic or systolic)).mp.	2464
44	36 or 37 or 38 or 39 or 40 or 41 or 42 or 43	2227587
45	35 or 44	3807739
46	3 and 17 and 45	4706
47	limit 46 to (english language and humans and yr="2000 -Current" and ("adult (19 to 44 years)" or "middle aged (45 plus years)") and journal article)	**1926**

Note: # 3 indicates records related to South Asia, #17 indicates records related to physical activity, # 45 indicates records related to chronic disease or their markers, # 46 indicates records related to South Asia and physical activity and chronic disease/markers

Table 2: Basic characteristics of the studies reviewed

Author	Year	Country	Study population	Sample size	Outcome	PA types	Quality rating
Quasem (1)	2001	Bangladesh, India	60 years and above	1203	HTN	Total PA	Fair
Zachariah (2)	2003	India	40-60 years	314	HTN	Occupational PA	Poor
Subburam (3)	2009	India	45-60 years	406	HTN	Occupational PA	Poor
Lloyd-Sherlock (4)	2014	India	50 years and above	7238	HTN	Total PA	Fair
Saeed (5)	2014	Afghanistan	40 years and above	1183	HTN	Walking	Fair
Snehalatha (6)	2003	India	20 years and above (specific results for 40 years and above)	11216 (not reported for ≥ 40 years)	T2D	Total PA	Poor
Ansari (7)	2009	Pakistan	45-64 years	2053	T2D	Total PA, LTPA, walking, cycling and household activities	Fair
Chhetri (8)	2009	Nepal	60 years and above	1633	T2D	LTPA	Fair
Jain (9)	2008	India	Men 40-60 years	394 (197 cases and controls each)	CHD	Total PA	Fair
Dhungana (10)	2015	Nepal	40 years and above	166	10 year CVD risk level (coronary heart disease or stroke) by WHO/ISH risk prediction chart	Total PA	Fair
Menon (11)	2016	India	18 years and above (results specific to 40-59 years)	4507 (2597 in 40-59 years)	10 years and lifetime CVD risk score	Total PA	Poor
Hamid (12)	2010	Pakistan	40 years and above	300 (150 cases and controls each)	Metabolic syndrome	Total PA	Fair
Gosh (13)	2005	India	Women 55 years and above	200	Percentage of Body fat, fat mass index and fat-free mass index	Walking	Fair

Kumar (14)	2008	India, Sri Lanka	48-69 years	330 (165 cases and controls each)	Total cholesterol and blood pressure	Walking	Poor
Bhatt (15)	2016	India	40-50 years	90	BMI, Waist circumference, fat mass and fat-free mass	Walking, cycling, PA level	Poor
Jahan (16)	2017	India	40-60 years	145	BMI, WC, WHR, blood pressure and metabolic syndrome	Total PA and Step count	Fair
Dey (17)	2009	India	Post-menopausal women	828 (431 cases, 397 controls)	BC	Total household PA	Fair
Mathew (18)	2009	India	Postmenopausal women	1659 (968 cases and 691 controls)	BC	Total household PA, walking, watching television (weekdays and weekends)	Fair
Keramat (19)	2008	India,Iran	Post-menopausal women	354 (203 cases, 151 controls)	Osteoporosis	Walking and LTPA	Fair
Lekamwasam (20)	2009	Sri Lanka	Men 50 years and above	1147	Osteoporosis	Total PA	Poor
Begum (21)	2014	Bangladesh	16-65 years (results specific for 46-65 years)	500 (189 in 46-65 years)	Osteoporosis	Total PA	Fair
Shetty (22)	2014	India	50 years and above	252	Osteoporosis	Total PA	Poor
Shenoy (23)	2017	India	41-60 years	1911	Osteoporosis	LTPA	Fair
Bishwajit (24)	2017	Bangladesh, India, Nepal, Pakistan, Sri Lanka	50 years and above	1189	Self-reported back pain	Total PA (vigorous and moderate) and walking	Poor

Table 3: Characteristics and results of studies: Cardiometabolic outcomes

Author (year)	Study design	Type of PA (recall period)	Measure of association	Results	Conclusion
Quasem (2001) (1)	Cross-sectional	TPA (on an average day)	Adjusted OR and 95% CI	Sedentary group: Ref<br>Mild PA: 0.66 (0.49-0.89)<br>Moderate PA: 0.54 (0.31-0.96)<br>Adjusted for BMI, diabetes mellitus, education, residence status, current smoking status, age, sex, marital status, religion, past history of smoking	Self-reported mild or moderate PA is associated with lower odds of HTN.
Zachariah (2003) (2)	Cross-sectional	OPA (regular day)	Adjusted OR and 95% CI	Sedentary group: Ref<br>Mild PA: NS<br>Moderate PA: 0.35 (0.13-0.94)<br>Adjusted for age, BMI, sex, self-reported diabetes mellitus, socioeconomic status and smoking	Prevalence of HTN was lower among participants involved in occupations requiring moderate or greater PA.
Subburam (2009) (3)	Cross-sectional	OPA (on an average day)	Adjusted OR and 95% CI	Effect estimates not reported	OPA is not associated with HTN.
Lloyd-Sherlock (2014) (4)	Cross-sectional	TPA	Adjusted OR and 95% CI	Low: 1.21 (1.03-1.43)<br>Moderate: 0.99 (0.83-1.17)<br>High: Ref	Higher odds of HTN among those with low PA.
Saeed (2014) (5)	Cross-sectional	Walking	Adjusted OR and 95% CI	Frequency of walking per week in hours<br><10 hours/week: Ref<br>10-30 hours/week: 0.66 (0.51-0.86)<br>≥30 hours/week: 0.55 (0.37-0.77)<br><br>Walking to workstation<br>No: 1.22 (0.96-1.55)<br><br>Adjusted values are not significant. Not reported in the paper.	Walking is not associated with HTN.
Snehalatha (2003) (6)	Cross-sectional	TPA	Adjusted OR and 95% CI	Effect estimates not reported	TPA is not associated with T2D.

Ansari (2009) (7)	Cross-sectional	TPA, LTPA, walking, cycling and household activities (last 2 years)	Adjusted HR and 95% CI	LTPA<br>Male: 0.78 (0.57-1.28)<br>Female: 0.72 (0.58-0.97)<br><br>TPA<br>Male: 1.0 (0.60-1.58)<br>Female: 0.75 (0.65-1.08)<br><br>Adjusted for age and BMI<br><br>Stair climbing inversely associated with T2D<br>Cycling: 0.82 (0.68-1.00)<br>Household activities and walking are not associated with T2D.	TPA is not associated with T2D among both men and women while LTPA is negatively associated with T2D among women. Stair climbing and cycling are also inversely associated with T2D.
Chhetri (2009) (8)	Cross-sectional	Exercise	Adjusted OR and 95% CI	Diabetes diagnosed during the survey<br>Yes: 1.26 (p-value: 0.175)<br><br>Diabetes diagnosed before the survey<br>Yes: 1.56 (p-value: 0.045)<br><br>Adjusted for age, education, health perception, caretaker at night, disturbed sleep, social participation, friends help in visiting health centre, family history of diabetes, history of HTN, family history of HTN, waist circumference	Exercise is only associated with the risk of diabetes diagnosed before the survey.
Jain (2008) (9)	Matched case-control	TPA (in a normal week)	Adjusted OR and 95% CI	Yes: Ref<br>No: 1.28 (0.82-2.00)	No statistically significant association existed between PA and CHD.
Dhungana (2015) (10)	Cross-sectional	TPA (in last 7 days)	p-value from chi-square test	<600 METs vs ≥600 METs: non- significant (p=0.219)<br>Unadjusted association	PA is not associated with CVD risk.
Menon (2016) (11)	Cross-sectional	TPA	p-value from chi-square test	High (>3000MET) vs Moderate (600-3000 METs) vs Low (<600 METs)<br>p= <0.001 (40-59 yrs) (0.009 for 60-79 yrs)<br>Unadjusted association	Decreased PA is associated with high 10-year CVD risk among 60 to 79 year age group.

<table>
<tr><td>Kumar (2008) (14)</td><td>Matched case-control</td><td>Brisk walking</td><td>P-value from student t-test</td><td><table>
<tr><td>Brisk walking</td><td colspan="2">Total cholesterol</td><td colspan="2">Blood pressure (systolic/diastolic)</td></tr>
<tr><td>≥180 mins/wk</td><td>168.34±8.67</td><td>183.36±4.52</td><td>117.52±3.39
76.46 ± 4.23</td><td>139.17±9.29
82.37 ± 4.73</td></tr>
<tr><td>≤180 mins/wk</td><td>156.48±7.48</td><td>168.34±7.24</td><td>101.23±4.19
70.16±5.62</td><td>126.18±912.83
79.12±3.67</td></tr>
</table></td><td>A significant difference existed in total cholesterol and blood pressure between those who walked for >180min/wk and less.</td></tr>
<tr><td>Gosh (2005) (13)</td><td>Cross-sectional</td><td>Brisk walking (in a normal week)</td><td>Standardised beta coefficients</td><td>Percentage of body fat (PBF): p<0.05
Fat mass index (FMI): p<0.05
Fat-free mass index (FFMI): p<0.01</td><td>Walking had a protective effect on PBF, FMI and FFMI.</td></tr>
<tr><td>Hamid (2010) (12)</td><td>Matched case-control</td><td>TPA</td><td>Adjusted OR and 95% CI</td><td>Yes: Ref
No PA: 2.47 (1.50-4.07)</td><td></td></tr>
<tr><td>Bhatt (2016) (15)</td><td>Cross-sectional</td><td>Walking, bicycling, physical activity level (PAL)</td><td>Correlation coefficients</td><td>Correlation coefficients
Walking: NS
Bicycling: -0.41(body weight), -0.25 (BMI), -0.39 (WC), -0.40 (fat mass), 0.31 (fat free mass)
PAL: -0.63(body weight), -0.59 (BMI), -0.57 (WC), -0.47 (fat mass), 0.45 (fat free mass)
All significant at 1% probability level</td><td>Walking had no effect but bicycling and PAL are negatively correlated with body composition measures (BMI, WC and fat mass) and positively with fat-free mass.</td></tr>
<tr><td>Jahan (2017) (16)</td><td>Cross-sectional</td><td>Walking (in last 7 days)</td><td>Correlation coefficients</td><td>Walking categorised as <5000 steps/day (sedentary), 5000-7499 steps/day (low active), 7500-9999 steps/day (somewhat active) and 10,000 to 12,499 steps/day (active)
BMI: p=0.273
WC: 0.685
Height circumference (HC): 0.632
WHR: 0.913
Systolic blood pressure (SBP): 0.05
Diastolic blood pressure (DBP): 0.502
No of MeS: 0.41</td><td>No significant correlation between steps per day and health indices except SBP.</td></tr>
</table>

Table 4: Characteristics and results of studies: Breast Cancer

Author (year)	Study design	Type of PA (recall period)	Measures of association	Results	Conclusion
Dey (2009)(17)	Matched case-control with 431 cases and 397 controls	Household PA (on a normal day)	Adjusted OR and 95% CI	ER+ cases vs. controls<br>PA <3hr/day: 1<br>PA 3-4hr/day: 1.37 (0.81-2.33)<br>PA 5-6hr/day: 0.60(0.36-0.98)<br>PA >6hr/day: 0.56(0.29-1.09)<br>P for trend=0.003<br><br>ER- cases vs. controls<br>PA <3hr/day: 1<br>PA 3-4hr/day: 0.92(0.57-1.50)<br>PA 5-6hr/day: 0.70(0.46-1.05)<br>PA >6hr/day: 0.60(0.35-1.03)<br>P for trend=0.009<br>Values adjusted for age, religion, education, SES score, BMI, age at menarche, menstrual status, parity, age at marriage and total duration of breastfeeding	Increased duration of household PA decreased the risk of BC among postmenopausal women.
Mathew (2009) (18)	Matched case-control study with 968 cases and 691 controls	Total household PA, walking, watching television	Adjusted OR and 95% CI	Total household PA<br>PA <3hr/day: 1<br>PA 3-4hr/day: 0.84(0.59-1.19)<br>PA 4-5hr/day:0.77(0.54-1.09)<br>PA 5-6hr/day:0.49(0.34-0.72)<br>PA >6hr/day: 0.51(0.35-0.73)<br>P for trend=0.00001<br><br>Walking (to buy things/errands)<br>Nil: 1<br><30 min: 0.44 (0.29-0.65)<br>30-59 min:0.77 (0.57-1.03)<br>≥60 min: 0.66 (0.46-0.95)<br>P for trend=0.00032	Increased duration of household PA decreased the risk of BC among postmenopausal women.

				Washing and carrying water were significant but cooking, cleaning the house, ironing clothes, working in the garden, watching TV during weekdays and weekends were not statistically significant<br><br>Values adjusted for age, centre, religion, marital status, education, socio-economic status, residence status, BMI, waist and hip sizes, parity, age at first childbirth and duration of breastfeeding	

Table 5: Characteristics and results of studies: Musculoskeletal conditions

Author (year)	Study design	Country	Type of PA (recall period)	Measure of association	Results	Conclusion
Keramat (2008) (19)	Matched case-control	India	Exercise, walking	Adjusted OR and 95% CI	Exercise<br>Yes: 0.40 (0.30-0.90) (adjusted for age)<br>All associations between osteoporosis and exercise, other exercises and regular walking are statistically non-significant when adjusted for age, height and weight.	Exercise had a protective effect on osteoporosis among post-menopausal women when only adjusted for age.
Lekawasam (2009) (20)	Cross-sectional	Sri Lanka	TPA (in a normal day)	p-value from chi-square test	PA categorised as very active (5-7 days/week), moderately active (3-4 days/week) and less active (<3 days/week).<br>P-value for trend: 0.001	High degree of PA is associated with higher BMD.
Begum (2014) (21)	Cross-sectional	Bangladesh	TPA (in last 7 days)	Adjusted OR and 95% CI	<120 min/week: Ref<br>≥120 min/week:<br>For lumbar spine: 1.35 (0.65-2.81)<br>For femoral neck: 0.76 (0.31-1.84)<br>For lumbar spine or femoral neck: 1.26 (0.61-2.63)	TPA is not associated with osteoporosis at any site.
Shetty (2014) (22)	Cross-sectional	India	TPA	Adjusted OR and 95% CI	No: Ref<br>Yes: 0.4 (0.12-0.9), $p<0.0001$	TPA has a protective effect on osteoporosis.
Shenoy (2017) (23)	Cross-sectional	India	LTPA (in last 7 days)	Adjusted OR and 95% CI	Per 10 unit increase in METs: 0.68 (0.66-0.71), $p<0.001$	Every 10-unit increase in TPA is negatively associated with the risk

						of osteoporosis.
Bishwajit (2017) (24)	Cross-sectional	Bangladesh	Vigorous TPA	Adjusted OR and 95% CI	Almost every day: Ref<br>Women: Few days/week: 1.41 (0.75-2.61), Almost never: 1.12 (0.48-2.60)<br>Men: Few days/week: 1.40 (0.93-2.09), Almost never: 1.42 (0.83-2.44)	Mixed associations existed between self-reported back pain and PA across gender and countries.
			Moderate TPA		W: Few days/week: 0.76 (0.48-1.20), Almost never: 0.86 (0.48-1.54)<br>M: Few days/week: 0.71 (0.48-1.04), Almost never: 0.77 (0.51-1.18)	
			Walking		W: Few days/week: 0.89 (0.56-1.41), Almost never: 0.93 (0.54-1.59)<br>M: Few days/week: 0.80 (0.49-1.29), Almost never: 0.78 (0.51-1.19)	
		India	Vigorous TPA		W: Few days/week: 0.89 (0.72-1.09), Almost never: 0.90 (0.65-1.24)<br>M: Few days/week: 0.76 (0.57-1.03), Almost never: 0.75 (0.45-1.25)	
			Moderate TPA		W: Few days/week: 1.36 (1.03-1.79), Almost never: 1.3 (1.04-1.69)<br>M: Few days/week: 1.38 (1.02-1.88), Almost never: 1.56 (1.00-2.44)	
			Walking		W: Few days/week: 1.26 (1.00-1.58), Almost never:: 1.33 (1.01-1.75)<br>M: Few days/week: 1.25 (0.91-1.69), Almost never: 1.43 (0.96-2.12)	
		Nepal	Vigorous TPA		W: Few days/week: 0.35 (0.88-1.13), Almost never: 0.55 (0.78-1.11)<br>M: Few days/week: 1.18 (0.78-1.77), Almost never: 1.02 (0.57-1.83)	
			Moderate TPA		W: Few days/week: 0.99 (0.99-0.70), Almost never: 0.67 (0.95-0.75)<br>M: Few days/week: 1.35(0.93-1.96), Almost never: 1.24 (0.67-2.28)	
			Walking		W: Few days/week: 0.90 (0.68-1.32), Almost never: 0.68 (0.19-1.59)<br>M: Few days/week: 1.36 (0.96-1.92), Almost never: 1.83 (1.14-2.93)	
		Pakistan	Vigorous TPA		W: Few days/week: 0.51 (0.25-1.05), Almost never: 3.72 (1.09–12.63)<br>M: Few days/week: 0.68 (0.41–1.13), Almost never: 1.33 (0.52–3.38)	
			Moderate TPA		W: Few days/week: 1.77 (0.76–4.09), Almost never: 2.49 (1.45–4.29)<br>M: Few days/week: 0.67 (0.45–0.99), Almost never: 1.33 (0.66–2.66)	
			Walking		W: Few days/week: 0.78 (0.52–1.17), Almost never: 0.61 (0.37–1.00)<br>M: Few days/week: 1.09 (0.68–1.72), Almost never: 2.85 (1.42–5.74)	
		Sri Lanka	Vigorous TPA		W: Few days/week: 1.62 (1.12–2.34), Almost never: 1.36 (0.79–2.32)<br>M: Few days/week: 1.37 (0.96–1.96), Almost never: 1.39 (0.89–2.18)	
			Moderate TPA		W: Few days/week:, 0.92 (0.62–1.36), Almost never: 1.58 (1.16–2.16)<br>M: Few days/week: 1.41 (0.97–2.03), Almost never: 1.17 (0.77–1.76)	
			Walking		W: Few days/week: 1.09 (0.76–1.57), Almost never: 0.86 (0.61–1.23)<br>M: Few days/week: 1.28 (0.83–1.96), Almost never: 1.13 (0.70–1.81)	

References

1. Quasem I, Shetye MS, Alex SC, Nag AK, Sarma P, Thankappan K, et al. Prevalence, awareness, treatment and control of hypertension among the elderly in Bangladesh and India: a multicentre study. Bulletin of the World health Organization. 2001;79(6):490.
2. Zachariah MG, Thankappan K, Alex SC, Sarma P, Vasan R. Prevalence, correlates, awareness, treatment, and control of hypertension in a middle-aged urban population in Kerala. Indian Heart Journal. 2003;55(3):245-51.
3. Subburam R, Sankarapandian M, Gopinath D, Selvarajan S, Kabilan L. Prevalence of hypertension and correlates among adults of 45-60 years in a rural area of Tamil Nadu. Indian Journal of Public Health. 2009;53(1):37-40.
4. Lloyd-Sherlock P, Beard J, Minicuci N, Ebrahim S, Chatterji S. Hypertension among older adults in low-and middle-income countries: prevalence, awareness and control. International Journal of Epidemiology. 2014;43(1):116-28.
5. Saeed KMI, Rasooly MH, Brown NJ. Prevalence and predictors of adult hypertension in Kabul, Afghanistan. BMC Public Health. 2014;14(1):386.
6. Snehalatha C, Ramchandran A, Kapur A, Vijay V. Age-specific prevalence and risk associations for impaired glucose tolerance in urban southern Indian population. Journal of Association of Physicians of India 2003;51:766-70.
7. Ansari RM. Effect of physical activity and obesity on type 2 diabetes in a middle-aged population. Journal of Environmental and Public Health. 2009;2009.
8. Chhetri M, Chapman R. Prevalence and determinants of diabetes among the elderly population in the Kathmandu Valley of Nepal. Nepal Med Coll J. 2009;11(1):34-8.
9. Jain P, Jain P, Bhandari S, Siddhu A. A case-control study of risk factors for coronary heart disease in urban Indian middle-aged males. Indian Heart J. 2008;60(3):233-40.
10. Dhungana R, Khanal M, Pandey A, Thapa P, Devkota S, Mumu S, et al. Assessment of Short Term Cardiovascular Risk Among 40 Years and Above Population in a Selected Community of Kathmandu, Nepal. Journal of Nepal Health Research Council. 2015;13(29):66-72.
11. Menon VP, Edathadathil F, Sathyapalan D, Moni M, Don A, Balachandran S, et al. Assessment of 2013 AHA/ACC ASCVD risk scores with behavioral characteristics of an urban cohort in India: Preliminary analysis of Noncommunicable disease Initiatives and Research at AMrita (NIRAM) study. Medicine. 2016;95(49).
12. Hamid N, Jillani G, Parveen N, Haq I, Arif S, H H. Metabolic Syndrome and its Relationship with associated risk factors J Med Sci. 2010;18(4):186-90.
13. Ghosh A, Das AC. Explaining body composition by some covariate factors among the elderly Bengalee Hindu women of Calcutta, India. The Journal of Nutrition, Health & Aging. 2005;9(6):403-6.
14. Kumar A, Sivakanesan R, Nagtilak S. Behavioral pattern, life style and socio economic status in elderly normolipidemic acute myocardial infarct subjects-a case control study from South Asia. Internet J Cardiovasc Res. 2009;6(2).
15. Bhatt N, Bains K, Aggarwal R. Association of selected anthropometric and body composition measures with diet and life style related factors among Punjabi adult males of India. Applied Biological Research. 2016;18(2):155-62.
16. Jahan N, Shenoy S. Relation of pedometer steps count & self reported physical activity with health indices in middle aged adults. Diabetes & Metabolic Syndrome: Clinical Research & Reviews. 2017;11:S1017-S23.

17. Dey S, Boffetta P, Mathews A, Brennan P, Soliman A, Mathew A. Risk factors according to estrogen receptor status of breast cancer patients in Trivandrum, South India. International Journal of Cancer. 2009;125(7):1663-70.
18. Mathew A, Gajalakshmi V, Rajan B, Kanimozhi VC, Brennan P, Binukumar BP, et al. Physical activity levels among urban and rural women in south India and the risk of breast cancer: a case–control study. European Journal of Cancer Prevention. 2009;18(5):368-76.
19. Keramat A, Patwardhan B, Larijani B, Chopra A, Mithal A, Chakravarty D, et al. The assessment of osteoporosis risk factors in Iranian women compared with Indian women. BMC Musculoskeletal Disorders. 2008;9(1):28.
20. Lekamwasam S, Wijayaratne L, Rodrigo M, Hewage U. Prevalence and determinants of osteoporosis among men aged 50 years or more in Sri Lanka: a community-based cross-sectional study. Archives of Osteoporosis. 2009;4(1-2):79-84.
21. Begum RA, Ali L, Akter J, Takahashi O, Fukui T, Rahman M. Osteopenia and osteoporosis among 16–65 year old women attending outpatient clinics. Journal of Community Health. 2014;39(6):1071-6.
22. Shetty S, Kapoor N, Naik D, Asha HS, Prabu S, Thomas N, et al. Osteoporosis in healthy South Indian males and the influence of life style factors and vitamin D status on bone mineral density. Journal of Osteoporosis. 2014;2014.
23. Shenoy S, Chawla JK, Gupta S, Sandhu JS. Prevalence of low bone health using quantitative ultrasound in Indian women aged 41–60 years: Its association with nutrition and other related risk factors. Journal of Women & Aging. 2017;29(4):334-47.
24. Bishwajit G, Tang S, Sanni Yaya ZF. Participation in physical activity and back pain among an elderly population in South Asia. Journal of Pain Research. 2017;10:905.

List of abbreviations

BMD	Bone Mineral Density
BMI	Body Mass Index
CHD	Coronary Heart Disease
CI	Confidence Interval
CVD	CardioVascular Disease
FFMI	Fat Free Mass Index
FMI	Fat Mass Index
HTN	Hypertension
LTPA	Leisure Time Physical Activity
MeTS	Metabolic Syndrome
METs	Metabolic Equivalents
MPA	Moderate Physical Activity
OR	Odds Ratio
PA	Physical Actvity
T2DM	Type 2 Diabetes Mellitus
WC	Waist Circumference
WHR	Waist-Hip Ratio
